# *CYR61* and *TAZ* Upregulation and Focal Epithelial to Mesenchymal Transition May Be Early Predictors of Barrett’s Esophagus Malignant Progression

**DOI:** 10.1371/journal.pone.0161967

**Published:** 2016-09-01

**Authors:** Joana Cardoso, Marta Mesquita, António Dias Pereira, Mónica Bettencourt-Dias, Paula Chaves, José B. Pereira-Leal

**Affiliations:** 1 Instituto Gulbenkian de Ciência, Oeiras, Portugal; 2 Ophiomics—Precision Medicine, Lisboa, Portugal; 3 Serviço de Anatomia Patológica, Instituto Português de Oncologia de Lisboa Francisco Gentil, E.P.E., Lisboa, Portugal; 4 Faculdade de Ciências da Saúde–Universidade da Beira Interior, Covilhã, Portugal; 5 Serviço de Gastrenterologia, Instituto Português de Oncologia de Lisboa Francisco Gentil, E.P.E., Lisboa, Portugal; University of Pennsylvania, UNITED STATES

## Abstract

Barrett’s esophagus is the major risk factor for esophageal adenocarcinoma. It has a low but non-neglectable risk, high surveillance costs and no reliable risk stratification markers. We sought to identify early biomarkers, predictive of Barrett’s malignant progression, using a meta-analysis approach on gene expression data. This *in silico* strategy was followed by experimental validation in a cohort of patients with extended follow up from the Instituto Português de Oncologia de Lisboa de Francisco Gentil EPE (Portugal). Bioinformatics and systems biology approaches singled out two candidate predictive markers for Barrett’s progression, *CYR61* and *TAZ*. Although previously implicated in other malignancies and in epithelial-to-mesenchymal transition phenotypes, our experimental validation shows for the first time that *CYR61* and *TAZ* have the potential to be predictive biomarkers for cancer progression. Experimental validation by reverse transcriptase quantitative PCR and immunohistochemistry confirmed the up-regulation of both genes in Barrett’s samples associated with high-grade dysplasia/adenocarcinoma. In our cohort *CYR61* and *TAZ* up-regulation ranged from one to ten years prior to progression to adenocarcinoma in Barrett’s esophagus index samples. Finally, we found that *CYR61* and *TAZ* over-expression is correlated with early focal signs of epithelial to mesenchymal transition. Our results highlight both *CYR61* and *TAZ* genes as potential predictive biomarkers for stratification of the risk for development of adenocarcinoma and suggest a potential mechanistic route for Barrett’s esophagus neoplastic progression.

## Introduction

Barrett’s esophagus (BE) is a premalignant metaplastic condition originated by the replacement of the normal squamous epithelium (NE) of the esophagus with a specialized columnar epithelial type that displays mixed gastric and intestinal characteristics [1]. BE is the major risk factor for the development of esophageal adenocarcinoma (EA) [2] and may progress to EA, through a low-grade to high-grade dysplasia (HGD) sequence. EA is the cancer with the fastest rising incidence in high-income countries [3] and has poor prognosis, with a high-related mortality and morbidity. Based on an estimated annual cancer risk of 0.5%, international guidelines universally recommend periodic endoscopic surveillance with a systematized biopsy protocol [4]. However, data reviewed on recent international guidelines estimates that BE risk of progression is very low (0.12%-0.33% patients/year [5, 6]. This fueled a running controversy on the costs/benefits of routine surveillance [7]. Apart from this debate, biopsy-based identification and grading of dysplasia in BE specimens is still the gold standard method to identify BE patients at risk of neoplastic progression [4], despite all the problems associated with such practice: costly, invasive, subjective dysplasia grading, biopsy sampling errors, unnecessary biopsying of low risk BE patients. Thus, a major current need in BE clinical management is of better methods and predictive biomarkers to stratify patients with an increased risk of disease progression [8], ideally early and/or biopsy-independent.

Recent high-throughput molecular studies have been instrumental for the enhanced understanding of many molecular events driving the BE "metaplasia-dysplasia-carcinoma" sequence [9, 10]. Despite all the resulting knowledge prompting the evaluation of >200 novel candidate biomarkers as predictors of progression (reviewed in [11]), none has yet reached routine clinical practice [12]. Molecular biomarkers of BE that predict progression to malignancy are still needed because their usage, alone or in combination with other biomarkers can facilitate more cost-effective surveillance. However due to the low progression rate of non-dysplastic BE few patients are available per discovery study. High-throughput molecular studies using robust sample sizes are scarce and thus published biomarker studies typically include very small patient cohorts. To maximize the discovery of new progression biomarkers, publicly available data needs to be used and combined into larger meta-cohorts. In particular the mining and re-analysis of existent gene expression data can be of great value, even if merging of distinct datasets may produce noisy predictions because such predictions can then be validated in patient cohorts.

In the present study, we set to define early molecular biomarkers predictive of BE progression to malignancy, through the usage of an innovative bioinformatics framework applied to publicly available global transcriptome data associated with BE to EA progression. *In-silico* generated prediction were validated in a cohort of patients under surveillance for more than ten years. We shown that *CYR61* and *TAZ* are up-regulated in BE index biopsies (negative for dysplasia) from patients that progress to cancer (P-BE), years before the development of EA as compared to index biopsies from BE patients that did not progress (non P-BE). To our knowledge, this is the first study to show that molecular changes associated with features of the epithelial to mesenchymal transition (EMT) invasive phenotype, usually detectable in late EA progression, can occur remarkably early in at risk BE mucosa. These changes are observable also at the protein level and show promise of clinical utility.

## Materials and Methods

### Public data collection, pre-processing and graphical display

We mined Gene Expression Omnibus (GEO) repository (http://www.ncbi.nlm.nih.gov/geo) [13, 14] or directly asked the authors for public microarray datasets on BE transcriptomes according to the criteria: 1) existence of clinical information on EA presence/absence at BE sample collection and 2) microarray experiments performed in the Affymetrix® Human Genome U133A microarray platform (HGU133a). Three datasets on HGU133a were retrieved: Kimchi *et al*. [15], Stairs *et al*. [16] and Watts *et al*. [17]. Kimchi *et al*. [15] study contained 8 BE samples adjacent to EA and thus these were classified as progressed BE (P-BE) plus 8 paired EA samples. Both Stairs *et al*. [16] and Watts *et al*. [17] series contained BE samples (n = 7 and n = 18, respectively) that were negative for dysplasia/EA at the time of collection and thus were classified as non-progressed (nonP-BE). In addition, the Watts *et al*. [17] dataset also contained EA samples but from distinct individuals of the nonP-BE samples.

Data analysis was performed with R Statistical Computing software [14] complemented with Bioconductor [18] packages. Heatmaps and Venn-diagrams were plotted using gplots (http://CRAN.R-project.org/package=gplots) and VennDiagram (http://CRAN.R-project.org/ package=VennDiagram) packages, respectively. Affy [19] and frma [20] packages were respectively used for raw data uploading and pre-processing and for frozen robust multi-array (fRMA) normalization. The R script used is available upon request.

### Differential expression analysis

We have used a Bayesian differential expression analysis (DEA) approach implemented in the R package limma [21] to define differentially expressed genes. Threshold for selection of differentially expressed probe sets was set to a B-statistic parameter Lods (already adjusted for multiple testing) ≥5 and a log2 ratio ≥ +0.58 or ≤- 0.58. The very conservative Lods>5 was based on DEA results between EA samples from Kimchi *et al*. [15] and Watts *et al*. [17] datasets, where no significant DE probe sets are expected, to control for inter-dataset variability noise.

### Barcode analysis

Probe set barcode values were calculated with the frma [20] and frma-associated hgu133abarcodevecs package, using the method described by McCall *et al*. [22, 23] (http://rafalab.jhsph.edu/barcode/). A probe set was defined as expressed (= 1) or non-expressed (= 0) in a given sample according to fRMA cutoffs. BE barcodes were filtered per dataset using very stringent criteria. A probe set was integrated in the dataset barcode if expressed in 100% or ≥ 75% in P-BE or nonP-BE samples, respectively. Group-specific barcodes were calculated by intersecting probe set IDs expressed in each dataset. EA-specific dataset barcodes were estimated as for nonP-BE i.e. probe sets were expressed in ≥ 75% samples to be integrated in the EA barcode.

### Gene set enrichment analysis

To find over-represented gene ontology biological processes (GO-BP) among specific sets of genes we used the gene set enrichment analysis (GSEA) tool from InnateDB (http://www.innatedb.ca/) using Entrez ID as gene identifier.

### GeneMANIA network analysis

The guilt-by-association GeneMANIA Cytoscape plugin algorithm [24] (http://genemania.org/) was used to identify genes functionally-related to our query genes, with gene symbols as identifiers.

### Samples and clinical data

For validation we used 19 formalin fixed paraffin-embedded (FFPE) BE samples from P-BE and nonP-BE (clinical data in S1 Table) of a cohort of 331 non-dysplastic Barrett’s esophagus enrolled in an endoscopic surveillance program (mean surveillance of 6.2 years ranging from 1 to 25) at the Instituto Português de Oncologia de Lisboa Francisco Gentil (IPOLFG), with an observed incidence of high-grade dysplasia or adenocarcinoma of 3,4/1000 patient-years during about the 30 years of existence of the surveillance program. All cases, at diagnosis, were analysed as per normal routine by two experienced GI pathologists. Patients select for this study were part of a cohort of patients diagnosed with Barrett’s Esophagus under surveillance at the IPO. This included a group of nine patients that progressed to high grade dysplasia or adenocarcinoma during surveillance (Progressed, P-BE), being the diagnostic confirmed by two independent pathologists, as standard practice and international recommendations. It included another group, as control, of 10 patients that did not display any dysplasia or carcinoma at any time during surveillance (termed non-Progressed, nonP-BE). In our cohort, progressor patients were defined as those with no dysplastic Barrett´s esophagus in the index endoscopy who progress during follow up to HGD or ADC. Non-progressors were defined as patients with no dysplastic Barret’s esophagus in the index endoscopy who remind free of dysplasia or ADC during a mean follow-up similar to that of progressors. The non-Progressed patients were randomly selected from our database. In the P-BE patients we analyzed samples from two time points, before and after malignant progression, named as t0 and t1. In the first time point (t0) we studied the initial biopsy diagnosed BE negative for dysplasia. In the second time point (t1) we examined two areas, the BE and the adjacent HGD/EA on mucosectomies or surgical pieces from these same patients. In the control group of nonP-BE patients we also studied two time points: t0 and t1 for the index and for most recent follow-up biopsies, respectively. In this set of patients all samples from t0, t1 or in any other follow-up archived sample (between t0 and t1) displayed any signs of malignancy. We chose to use a balanced number of non-progressors to avoid artificially inflating p-values in the comparisons. Histopathological characterization and area selection was carried out on hematoxylin- and eosin-stained sections under the supervision of an experienced pathologist. The study was approved by the institutional review board and a waiver of consent was obtained prior to initiating this retrospective study (project GIC/721 IPOLFG, EPE).

### RNA extraction and cDNA synthesis

Archived BE FFPE tissue sections (5 μm) were deparaffinized and counterstained with Mayer’s hematoxylin and eosin. BE-enriched areas were needle microdissected under the pathologist guidance. Total RNA was extracted with the RNeasy FFPE kit (Qiagen), according to manufacturer’s instructions with a slight modification: proteinase K cell-lysis at 56°C was performed overnight. The RNase-Free DNase Set (Qiagen) “on column” DNA digestion procedure was included. Each extracted RNA was reverse-transcribed with the First-Strand cDNA Synthesis kit (GE Healthcare), using a 1:1 mixture of random primers (pd(N)_6_) and oligo-dT primers (NotI-d(T)_18_. High quality total RNA (3 μg) from two control cell lines (HCT116 and a primary skin fibroblasts) was used to synthesize cDNA to be used as dilution standards in qRT-PCR.

### Quantitative real-time PCR

RNA concentration and integrity could not be assessed using standard methods due to known FFPE degradation issues and to the small amounts of extracted samples. Thus, to indirectly check the amount of each isolated total RNA FFPE sample and its quantitative real-time PCR (qRT-PCR) downstream performance, we prepared two standards dilution series using cDNA from the two control cell lines, corresponding to 100, 10 1, 0.1, 0.01, 0.001 and 0.0001 ng of the original total RNA. These series were subsequently used to calculate a qRT-PCR standard curve for the non-differentially expressed gene *MAPKAPK2* (Lods = -2.7). Primer sets were designed with the NCBI Primer-BLAST tool [25], to work at 59°C and with an amplicon length of 70–100bp (S2 Table). Duplicates of each BE sample were analyzed by qRT-PCR using SsoFast™ EvaGreen® Supermix (Bio-Rad, Hercules CA, USA) reagent in 10μL of reaction mixture containing template (2μL, ~200pg/μL) and primers (0.5μM each). Samples were processed in a CFX96 Touch™ Real-Time PCR Detection System (Bio-Rad, Hercules CA, USA) according to the cycling program: 95°C for 60 s, 50 cycles of 95°C for 10s and 59°C for 15s. Fluorescence data collection occurred at 59°C. Relative differential expression analysis of target genes by qRT-PCR was based on the 2^-ΔΔCt^ methodology from Livak *et al*. [26] using mean quantification cycle of duplicates as cycle threshold (Ct) compared to the Ct of the calibrator gene GAPDH.

### Immunohistochemistry

Immunohistochemistry (IHC) of BE samples (3 μm thick tissue sections) was performed according to standard protocols. Primary antibodies were diluted in Bond Primary Antibody Diluent (Leica Microsystems) plus background-reducing components at the dilutions: *CYR61* (1:600, mouse monoclonal [3H3], Abcam, ab80112), *TAZ* (1:300, mouse monoclonal, Abcam, ab118373), *E-Cadherin* (1:80, mouse monoclonal 4A2C7, Invitrogen, 33-4000). Antigen retrieval consisted of pressure-cooking for 6 minutes in pH6 sodium citrate 0.01M buffered solution for *CYR61*, of 20 minutes of microwave exposure at 750W in pH6 sodium citrate 0.01M buffer and of 25 minutes in Epitope Retrieval Solution 2 (ER2) Leica Bond III system for E-Cadherin. Signal detection of *CYR61* and *TAZ* was obtained using the Rabbit/Mouse Peroxidase/DAB+ Dako REAL Envision Detection System while *E-Cadherin* visualization was performed in the Leica Bond III system with the detection system Bond Polymer Refine Detection plus Bond DAB Enhancer. Nuclei were counterstained with Mayer’s hematoxylin. Images were acquired on a Leica DM5500 microscope.

IHC staining specificity was evaluated with a three level score. IHC scores for CYR61 antibody (ab80112) were defined as “Low” when a weakly positive diffuse protein staining was observed (+), “Intermediate” when scored areas included few areas of weakly positive and most areas with positive diffuse staining (++) and “High” when no negative areas were observed and the majority of evaluated areas presented a strongly positive diffuse staining (+++). IHC scores for TAZ antibody (ab118373) were defined as “Low” when protein staining was negative to weakly positive cytoplasmatic diffuse staining in the majority of visualized areas (-), “Intermediate” when weakly positive cytplasmatic and low to strong nuclear staining was observed in some areas (+) and “High” when low to strong cytoplasmatic staining and very strong nuclear staining was observed in the majority of evaluated areas (++).

### Western-blotting

The western-blotting procedure was performed according to standard protocols. Total protein extracts (20μg) from the two breast cancer cell lines MDA231 and MCF7 were resolved by SDS-PAGE on a 12% acrylamide gel (BIO-RAD) and transferred to a PVDF membrane (GE Healthcare) on a Mini-Protean system (BIO-RAD). The molecular weight marker used for electrophoresis was the Kaleidoscope (∫BIO-RAD) and transfer conditions were 250 mA, 100 min. CYR61 (ab80112) and TAZ (ab118373) antibodies were both diluted 1:200 in 0.2% fish skin gelatin, 1x TBST and the loading control β-Actin antibody (Sigma) was diluted 1:2000 in 1% milk, 1x PBS. All primary antibodies were incubated overnight at 4°C. Detection for all antibodies was performed by incubation with mouse Horseradish Peroxidase antibody (Jackson Immunoresearch) at 1:10.000 dilution and the western-blot signal developed with and ECL system (BIO-RAD) and detected on an x-ray Amersham Hyperfilm ECL (GE Healthcare).

### Statistical analysis

Data analysis was performed with R language for Statistical Computing [14]. Expression differences between P-BE and nonP-BE microarray data was determined with a Bayesian T-test implemented in the R package limma [21]. We used hypergeometric testing to assess gene set enrichment. Statistical significance of qRT-PCR data was calculated with Wilcoxon Rank Sum test (confidence level = 0.95). IHC categorical data was analyzed with Pearson’s Chi-squared test.

## Results

### Hypotheses generation: mining public gene transcriptomics data

**Fig 1:**
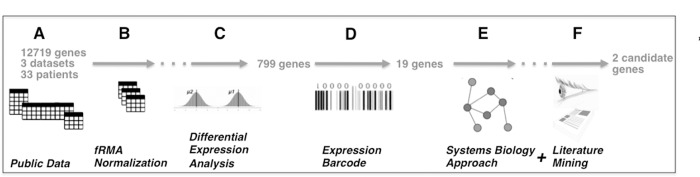
Bioinformatics analysis workflow of BE datasets for biomarker discovery. **A.** Three publicly available microarray datasets of BE data containing 33 BE samples with progression information were used to interrogate the expression levels of 12719 unique genes. **B.** After data normalization with frozen robust multi-array (fRMA) we first performed **C.** differential gene expression analysis, which allowed the identification of 799 differentially expressed genes. Normalized fRMA data was subsequently submitted to **D.** gene expression barcode which further restricted the number of selected candidates to 19. The combined usage of a **E.** systems biology approach plus **F.** manual literature curation selected the two most promising candidates.

We implemented a bioinformatics pipeline for data mining that takes as input the small number of expression profiling data sets available for nonP-BE, P-BE and EA (Fig 1A), insures that data is comparable using fRMA normalization (Fig 1B) and identifies genes differentially expressed between P-BE and nonP-BE (Fig 1C). Subsequently cross checks these against a database of human gene expression patterns to binarize the genes with bimodal gene expression (Fig 1D) and implements a network analysis to cross check the selected list of candidate genes against other data types (such as protein interactions, gene co-expression, etc) for plausibility (Fig 1E). Finally, the output of this pipeline is submitted to one additional filtering step, based on manual literature curation of selected genes (Fig 1F, Table 1). From a list of 12749 unique starting genes, our approach predicts the two genes *CYR61* and *WWTR1* (alias *TAZ*) that *in silico* can distinguish P-BE from nonP-BE samples. Details of the methods are given in methods section, and a detailed step-by-step description of each step and resulting lists of genes is given as supplementary material (S1 Fig and S1 Results).

**Table 1:** Cancers where *CYR61* and *TAZ* over-expression has been previously correlated with poor outcome.

Cancer Type	*CYR61 (references)*	*TAZ (references)*
**Breast**	[27]	[28, 29]
**Prostate**	[30–32]	---
**Colorectal**	[33]	[34]
**Gastric**	[35, 36]	---
**Esophageal Squamous Cell**	[37, 38]	---
**Esophageal Adenocarcinoma**	[39]	---
**Pancreas**	[40, 41]	---
**Hepatocellular**	[42]	---
**Non-Small Cell Lung**	[43]---	[43],[44]
**Thyroid Carcinoma**	[45]	[46]ª
**Renal cell carcinoma**	[47]	---
**Ovary**	[48]	---
**Glioma**	[49]	[50]
**Osteosarcoma**	[51]	---
**Epithelioid Hemangioendothelioma**	---	[52]
**Oral (squamous cell)**	[53, 54]	---

ªPapillary

### mRNA levels of *CYR61* and *TAZ* distinguish nonP-BE from P-BE in paraffin-embedded samples

Given the existing variability associated with microarray technology results (lab-, user- platform-associated, etc) and the technology limitations (probe sensitivity and specificity) it is essential to use an independent mean to verify and reproduce the results of candidate genes.

We evaluated *CYR61* and *TAZ* as early biomarkers of BE progression in a validation set of FFPE samples from 19 BE patients (detailed characteristics of the patients used in the validation set of the present study is available in S1 Table). In total, 9 P-BE patients (t0, n = 9 and t1, n = 9 samples) and 10 nonP-BE patients (t0, n = 10 and t1, n = 10 samples) were included (see Materials and Methods)

**Fig 2:**
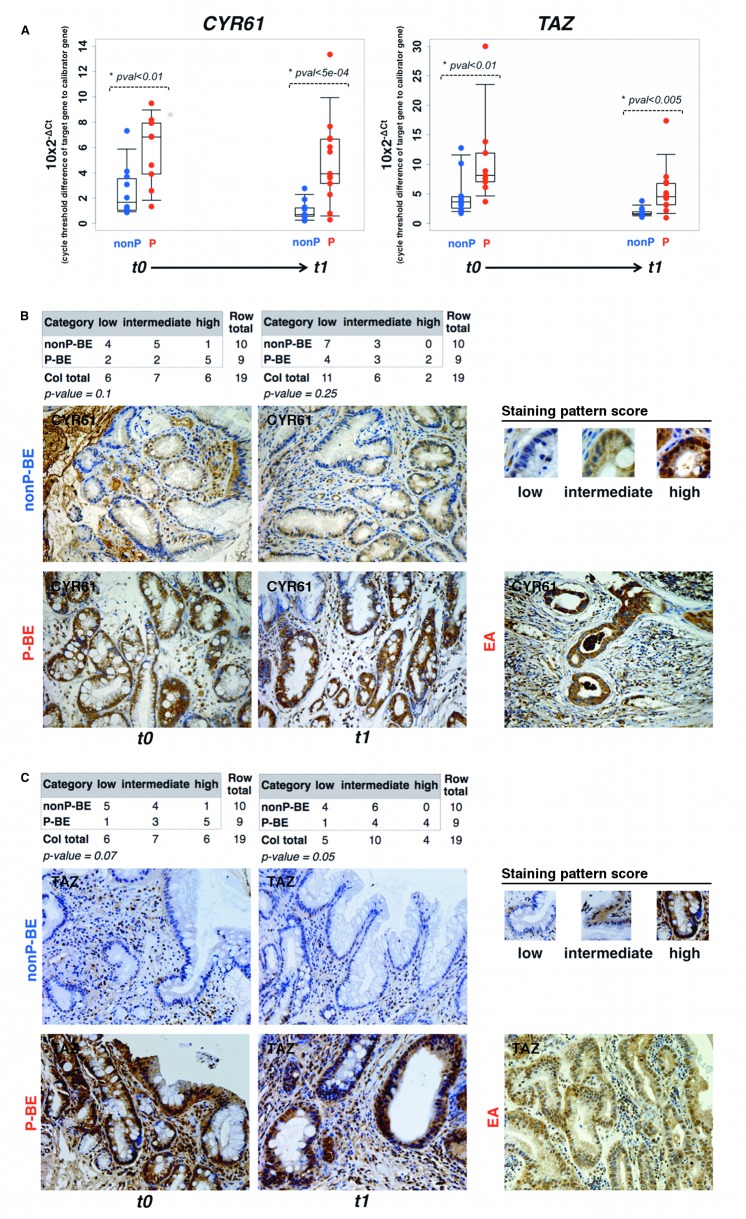
*CYR61* and *TAZ* mRNA and protein levels are significantly increased in early and late at risk BE biopsies. **A.** Timeline analysis of *CYR61* (left panel) and *TAZ* (right panel) expression levels by qRT-PCR of P-BE associated with EA (t1) and in the patient-matched BE index biopsies, free of dysplasia/EA (t0). Index BE biopsies were collected at t0 while, after several years of follow-up, EA-associated BE biopsies in the P-BE group and EA-free BE biopsies in the nonP-BE group were designated as collected at t1. The average years of follow-up between t0 and t1 was 4.6 and 9.4 years for P-BE and nonP-BE samples. **B.** and **C.** panels display respectively representative samples of *CYR61* and *TAZ* protein levels in BE (t0 and t1) and in EA, evaluated by immunohistochemistry. Staining patterns used to score the protein levels (low, intermediate and high) are represented on the top right of each panel. The counts and statistical test (Pearson’s Chi-squared test) results are represented in the top left of the panels. (Magnification ×200).

Using qRT-PCR we compared *CYR61* and *TAZ* mRNA levels from t1 P-BE tissue co-occurring with HGD/EA with nonP-BE samples without any histological signs of malignancy. The analysis revealed that the transcriptional levels of both genes were significantly increased (*P* value<0.005) in P-BE samples (Fig 2A) as predicted *in silico*. We have also detected a significant up-regulation (average fold change >2, *P* value <0.01) in the index samples (t0) of P-BE patients, years before the development of HGD/EA as compared to nonP-BE index samples (t0) from patients that never developed HGD/EA (Fig 2A, Table 2). This early up-regulation could be detected as early as 13 years (average: 4.6 years; range: 1-13 years) in the P-BE group. In the nonP-BE group, the maximum follow-up interval was of 17 years (average: 9.4 years; range: 3-17 years). In addition, using the microarray and qRT-PCR data we verified that *CYR61* and *TAZ* expression levels are not correlated and thus P-BE and nonP-BE samples could be better segregated when using independent information from both markers (S4 Fig). Their combined usage may enhance sensitivity for the early detection of patients at risk of BE malignant progression in BE index samples.

**Table 2:** Per patient quantitative assessment of *CYR61* and *TAZ* expression levels by qRT-PCR and IHC.

		*Age at*			*t0*				*t1*			
		*(biopsy order)*		*YearsFUp*	*qRT-PCR*		*IHC*		*qRT-PCR*		*IHC*	
*Group*	*ID*	*1*^*st*^	*last*		*CYR61*	*TAZ*	*CYR61*	*TAZ*	*CYR61*	*TAZ*	*CYR61*	*TAZ*
nonP-BE	11	64	81	17	Low	Low	++	+	Low	Low	+	+
nonP-BE	13	62	65	3	Low	Low	++	+	Low	Low	+	+
nonP-BE	14	65	75	10	Low	Low	++	++	Low	Low	+	+
nonP-BE	21	66	77	11	Low	Low	+	-	Low	Low	++	-
nonP-BE	22	67	74	7	Low	Low	+	+	Low	Low	+	+
nonP-BE	25	32	42	10	High	High	++	-	Low	Low	+	-
nonP-BE	26	64	76	12	Low	High	+	-	Low	Low	++	+
nonP-BE	27	42	52	10	Low	Low	++	-	Low	Low	++	+
nonP-BE	28	52	63	11	Low	Low	+++	-	Low	Low	+	-
nonP-BE	29	51	54	3	Low	Low	+	+	Low	Low	+	-
P-BE	2	71	72	1	Low	High	+++	+	Low	High	+++	+
P-BE	3	46	59	13	High	High	++	++	Low	High	+	++
P-BE	4	50	54	4	Low	High	+++	+	Low	Low	++	+
P-BE	5	51	53	2	Low	High	++	++	Low	Low	+	++
P-BE	6	45	48	3	High	High	+++	+	High	High	++	-
P-BE	17	62	67	5	High	High	+++	++	High	High	+	+
P-BE	18	48	54	6	High	High	+	-	High	High	+++	+
P-BE	19	68	69	1	Low	Low	+++	++	High	Low	+	++
P-BE	20	51	57	6	High	High	+	++	Low	Low	++	++

Degree of immunostaining is indicated by the “+” and “-”signs: “+++” = very strong staining; “++” = strong staining; “+” = weak staining; “-”= absence of positive staining. YearsFUp = Years of follow-up; qRT-PCR = quantitative real time PCR; IHC = immunohistochemistry.

### Protein levels of *CYR61* and *TAZ* distinguish P-BE from nonP-BE in paraffin embedded samples

Validation of the *in silico* predictions by qRT-PCR is encouraging, but clinical use could be simpler if routine techniques such as immunohistochemistry were to be used. We have thus implemented an IHC assay. We have started by evaluating the specificity of both antibodies by Western-blot analysis (S5A Fig). IHC results of *CYR61* and *TAZ* proteins were categorized into three different groups according to staining intensities (Fig 2B, Table 2): low, intermediate and high. CYR61 antibody presented a diffuse cytoplasmatic and/or nuclear staining as shown in the positive control (S5B Fig) and TAZ antibody mostly stained nuclei although it is also presented a diffuse pattern in the cytoplasm (S5C Fig). Blinded analysis showed that despite the somewhat heterogeneous cytoplasmatic staining of *CYR61* protein, its levels were mainly intermediate to high in the P-BE group and mostly varied from low to intermediate in the nonP-BE group (Fig 2B). *CYR61* protein over-expression differences were more pronounced in the early time point t0. Interestingly, samples displaying the highest amounts of *CYR61* often exhibited strong nuclear accumulation. As for *CYR61*, despite some heterogeneity of *TAZ* IHC pattern (Fig 2C, Table 2), *TAZ* protein levels were increased in the P-BE as compared to nonP-BE group, with P-BE samples from t0 displaying a more distinct *TAZ* over-expression. Overall, protein levels validated the *in silico* transcriptional changes and correlated with qRT-PCR results (Table 2). Further, they highlighted once more the very early (at t0) differences of *CYR61* and *TAZ* expression between progressors and non-progressors.

### *CYR61* and *TAZ* up-regulation is correlated to an epithelial-to-mesenchymal transition phenotype

**Fig 3:**
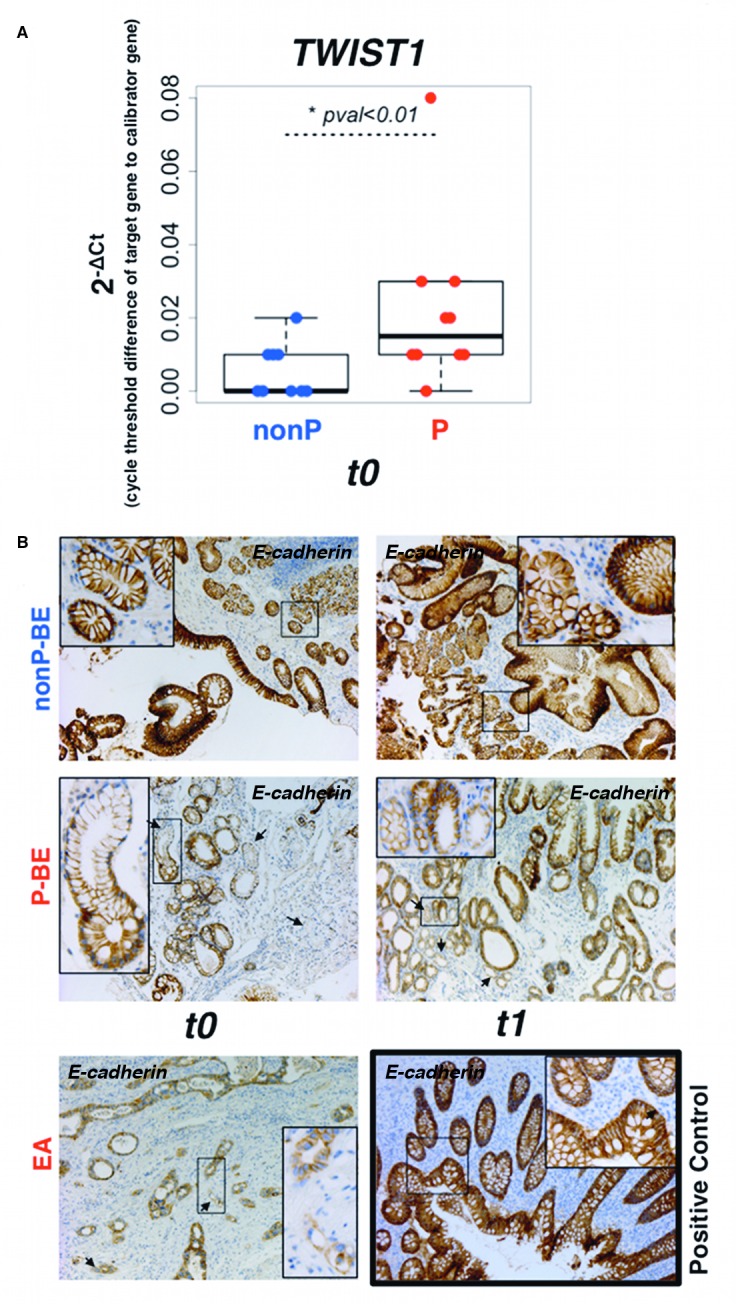
Changes in epithelial-to-mensenchymal biomarkers are visible in early and late BE backgrounds. **A.** qRT-PCR validation of *TWIST1* transcription factor in the patient-matched BE index biopsies, free of dysplasia/EA (t0). **B.** *E-cadherin* protein levels were evaluated by immunohistochemistry staining in P-BE associated with EA (t1) and in the patient-matched BE index biopsies, free of dysplasia/EA (t0). Arrows denote foci of lower *E-cadherin* expression. Normal appendix was used as *E-cadherin* immunostaining positive control. (Magnification: picture ×100, detail ×200).

In our network analysis for prioritizing genes (Fig 1E), we found that EMT and stemness-related genes are significantly over-represented in the *in silico* P-BE samples (p = 5.5×10^-8^). Out of the 19 barcoded genes, 52% (*SPARC*, *CYR61*, *JUN*, *ACTN1*, *COL4A1*, *PPAP2B*, *DUSP1*, *CTSB* and *TAZ*) have been previously detected in molecular signatures of EMT/stemness phenotypes [55–57]. Such phenotypes are clearly associated with an aggressive clinical behavior, poor outcome and resistance to treatment (reviewed in [58]). Given the involvement of *CYR61*, *TAZ* and other P-BE up-regulated genes in stemness/EMT-related cellular functions (e.g. cell adhesion/motility, inflammation, differentiation/wounding, extracellular matrix) (reviewed in [58]) we checked if core EMT markers such as *TWIST1*, *ZEB1*, *SNAI1*, *SNAI2* and *CDH1* (alias *E*-*cadherin*) (reviewed by [59]) were also differentially expressed. *TWIST1* was the only significantly over-expressed gene (Lods = 5.84, fold change>2) in P-BE samples. As shown in Fig 3A, we validated *TWIST1* up-regulation by qRT-PCR analysis in early P-BE samples (t0) before the emergence of any microscopic signs of malignancy. Furthermore, using routine pathology IHC for *E-cadherin* we also detected foci of lower *E-cadherin* expression in P-BE samples both in early (t0) and late (t1) P-BE samples (Fig 3B), an observation usually associated with invasive EA cells. The appearance of such foci is indicative of very early P-BE cellular adhesion and/or extracellular matrix changes absent in nonP-BE samples. This observation suggests that an aggressive mechanism, typical of advanced metastatic lesions, is active in non-malignant BE cells of at risk patients. These features of neoplastic progression occur in P-BE in a time point far earlier than we anticipated. The presence in P-BE samples of alterations typical of aggressive behavior in the context of cancer, suggest that at very early stages in BE there is already a proneness for later development of dysplasia and EA.

## Discussion

In the present work, we aimed at maximizing the identification of potentially translatable biomarkers of early BE progression to EA using an original bioinformatics pipeline applied to public BE expression profiling data. This discovery framework allowed the straightforward comparison of BE samples from distinct datasets and the trimming of promising over-expressed biomarkers to *CYR61* and *TAZ* genes. Validation with qRT-PCR and IHC emphasized *CYR61* and *TAZ* over-expression as early markers of at risk BE index samples, years before HGD/EA emergence. The access to Barrett’s patients that progressed during the surveillance program allowed the unique opportunity to validate biomarkers in Barrett’s samples from the same group of patients before and after malignant progression. This allowed us to overcome the limitations derived from inter-patient variability in studies where different sets of patients were used in each step of Barrett’s malignant progression. To our knowledge, this is the first time that risk stratification biomarkers were validated in BE samples coming from the same set of patients. Finally, changes in EMT biomarkers were detected in index samples of at risk BE. This observation fits into *CYR61* and *TAZ* functional context and further suggests that in at risk BE, proteins associated with EMT can be operational from very early, before any visible signs of malignancy.

*CYR61* and *TAZ* emerged from our pipeline as the most promising biomarkers of at risk BE and we experimentally validated their up-regulation in a different cohort. Several studies have implicated *CYR61* and *TAZ* in the biology of major cancers (Table 1). BE is a metaplastic response of the esophageal surface to chronic injury caused by gastric reflux, possibly amplified by an inflammatory response [60]. BE progression to EA could plausibly be mediated by the known functions of these two genes in extracellular matrix, cell migration, angiogenesis and stemness/EMT. *CYR61* is an important factor in acid-induced esophageal epithelial transformation [61] and its up-regulation is an early response of esophageal cells exposed to low pH [62]. In a non-esophageal context, *CYR61* plays a role in inflammation, it is notably expressed at wounded tissues [63] and also up regulated upon mechanical stress (reviewed in [64]). *CYR61* has also an independent prognostic value in esophageal squamous cell carcinoma [37, 38] and is a negative predictor in early onset sporadic colorectal [65, 66] and ovarian [48] cancers. Functionally, *CYR61* is a ligand for several integrins which in turn can trigger cancer cells motility (reviewed in [67]) as it was recently demonstrated in pancreatic cancer [41]. *TAZ* is a key mediator of mechanotransduction, also implicated in human tumorigenesis: *TAZ* transducer activities are required to sustain self-renewal and tumor-initiation capacities [29], cell proliferation and EMT in breast cancer stem cells [28, 68] and to regulate mesenchymal differentiation in malignant gliomas [50].

Many putative biomarkers of BE malignant progression resulted from previous studies (reviewed in [11]) but no biomarker has been used in routine clinical practice [12]. Typically, most biomarkers were discovered in a context of a detectable high-grade dysplasia/EA, a time point where the neoplasia is already established and therefore where markers of tumor development are of little use and only cancer progression is at stake. This type of cancer-associated molecular alterations in non-cancer tissues (e.g. BE and NE) but adjacent to a tumor are often referred to as a cancer field effect and have already been described in BE [69]. We analyzed *CYR61* and *TAZ* levels in non-dysplastic/EA-free BE index biopsies of P-BE and nonP-BE patients by qRT-PCR to distinguish whether *CYR61* and *TAZ* up-regulation were a cancer field effect or an early property of P-BE samples. We found that, in fact, the up-regulation of these genes in BE years before the appearance of dysplasia (t0), reveals the establishment of a signaling pathway prone for progression to dysplasia/EA at very early stages through an intrinsic alteration of cell properties that directly, or by interaction with stromal tissue will facilitate tumor initiating features. Interestingly, we observed that *CYR61* and to a lesser degree also *TAZ* expression levels, slightly decreased when progressing from index (t0) to advanced (t1) BE. Differences between P-BE and nonP-BE are thus more significant at t0. This phenomenon of increased expression on localized benign disease and a decreased expression upon progression to metastasis is known to occur in several contexts such as for *CYR61* in prostate cancer (reviewed in [70]). While no mechanistic explanation justifies yet this observation in BE, it is possible that, as in prostate, *CYR61* and *TAZ* are more important in the neoplastic initiation than progression.

Although *CYR61* up-regulation was previously described in BE samples where dysplasia/EA is already present [39], our work is the first to describe that such up-regulation is in fact a very early event in Barrett’s tumorigenic process, being an indicator for a later establishment of dysplasia/EA, since it is detected in BE index biopsies. *CYR61* belongs to the CCN family of six structurally related proteins, a multi-tasking group of secreted proteins which primarily function in adhesion, migration, proliferation, ECM synthesis, inflammation and mechanical stress regulation (reviewed in [67]). Furthermore, *CYR61* has an already established role in cancer malignant progression and prognosis in major and diverse tumors (Table 1) and is a downstream target of *TAZ* [71]. *TAZ* is a major downstream effector and is regulated by the Hippo tumor suppressor pathway, a pathway relevant in organ size control, tissue regeneration, stem cell self-renewal (reviewed by [72]), cell polarity and cancer (reviewed by [73]). *TAZ* has also been implicated in the malignant phenotype of several tumors (Table 1) but differently from *CYR61*, *TAZ* over-expression has never been under scrutiny in human BE or EA. We anticipate that the biological functions of both genes, *TAZ* as a major regulator of the hippo pathway and its downstream effector *CYR61*, may contribute to BE progression to EA and we demonstrate for the fist time that they have predictive value.

The functional context of our validated targets and of other genes detected in our analysis suggested the early occurrence of mechanisms known to operate in EMT. As additional examples of such, we detected early *TWIST1* up-regulation and lower *E-cadherin* expression foci very early in BE (i.e. in BE index samples) from patients that progressed later on to cancer. These are classical biomarkers associated to aggressive features of malignant progression such as EMT. The early occurrence of *in vivo* EMT in the absence of any histological signs of cancer was recently demonstrated in pancreatic cancer and can be partially facilitated by inflammation [74]. EMT occurs both in wound healing and tumors (reviewed in [75]) a scenario that fits into *CYR61* and *TAZ* functional context. How EMT could contribute to early stages of BE malignant progression and/or if only some EMT-related pathways are activated is currently unknown. However, EMT occurrence was described in EA [76] and in an immortalized normal epithelial cell line. In the latter *CYR61* up-regulation was critical and exacerbated acid-induced EMT phenotypes such as triggering *E-cadherin* loss [61]. Moreover, it was recently reported that *TAZ* and *CYR61* were implicated in lung cancer progression and EMT via angiomotin [43].

Our observations indicate that in particular, being *CYR61* an extracellular matrix secreted protein it harbors the potential to become a serum biomarker to stratify the risk of progression to malignancy in BE [32, 36] allowing for less invasive follow-up exams. This could supply an extra tool to define the risk of malignant progression and the possibility of reducing the number of biopsies needed, in particular for low risk patients.

Despite the very low BE progression frequency, we had access to a small, yet precious and very rare, cohort of FFPE follow-up samples for validation and more importantly to implement *CYR61* and *TAZ* detection by IHC, a method directly translatable to all pathology labs. Although FFPE-based qRT-PCR *CYR61* and *TAZ* measurements are a valid profiling option, already validated in oncologic diagnosis (reviewed in [77, 78]), assaying mRNA in the clinical routine may not be the most desirable because of technical and cost constrains. Since IHC is a method not limited by the quantity of material and can assess protein presence at single cell level, we tested commercially available antibodies against our two gene candidates and found two that, after optimization, the expected immunostains were obtained and the results could be easily translated to a routine diagnostic lab. Though the qRT-PCR and IHC data showed independently a significant difference between P-BE and nonP-BE samples in both timepoints, we have not observed clear correlation between the results of both methodologies within the same patient. The discrepant results observed in Table 2 may be justified do to inherent differences between qPCR and IHC, two techniques that are very different in nature. qPCR is quantitative, whether IHC is qualitative; furthermore, for the qPCR analysis we enriched the sample by microdissection, whereas no equivalent procedure was possible for IHC. In addition, IHC is prone to variation-dependent biases while qPCR is not observer dependent.

Given the very low BE progression frequency we will always be limited by the number of available archived collections of P-BE index samples (t0) and its follow-up samples until histological signs of malignancy are displayed (t1), which spans periods of many years. Additionally, our country has lower BE incidence rates when compared to other developed countries and we do not have a national BE register. Despite all this, our small cohort of patients that progressed during surveillance reproduces the actual expected risk progression in Barrett’s patients. Indeed, compared with the several international cohorts reviewed by the British Society of Gastroenterology guidelines [79] our cohort is quite representative. We are aware that larger numbers are needed in future tests with these two markers, which will certainly implicate several international multi-institutional collaborations.

## Conclusions

Given the running debate on the costs/benefits of BE surveillance programs [80–82] and despite the diminished risk of progression [83] biomarkers to better stratify the patients who have real increased risk of neoplastic progression are required. Furthermore, it is important to stress that the risk may be low but still, BE is the precursor lesion of one of the fastest growing cancer types in developed countries over the past decades. Our results support the use of *CYR61* and *TAZ* as early biomarkers to discriminate which BE patients have an increased risk to progress to dysplasia and cancer and further suggest that proteins/genes involved in EMT are critical to trigger the BE lesions that evolve to more aggressive lesions. Our findings also highlighted that these and other yet unknown markers should be supervised right from the non-dysplastic BE index biopsy. Such procedure has the potential to greatly improve BE management by sparing low risk patients from unnecessary invasive exams and to impact the overall costs (ethical and economical) of surveillance programs.

## Supporting Information

S1 Fig An original bioinformatics prioritization framework allowed the identification of at risk BE biomarkers. (PDF)

S2 Fig Microarray data of BE samples from distinct datasets is highly correlated. (PDF)

S3 Fig Differential expression analysis of P-BE and nonP-BE microarray data highlighted more than 700 genes potentially involved in BE malignant progression. (PDF)

S4 Fig CYR61 and TAZ expression levels are not correlated. (PDF)

S5 Fig Validation of CYR61 and TAZ antibodies specificity. (PDF)

S6 Fig GeneMania network of the 19 selected candidate biomarkers, network neighbors and associated function showed an enrichment of genes related with cell adhesion, motility and response to stimuli. (PDF)

S7 Fig CYR61 and TAZ are biological linked and share important cellular functions. (PDF)

S1 Results Systems biology approach for biomarker prioritization. (PDF)

S1 Table Clinical data of patients used for the validation set. (XLSX)

S2 Table Primer sequences for target and reference genes. (XLSX)

S3 Table Differentially expression analysis filtered genes (Lods > 5). (XLSX)

S4 Table Barcode genes exclusively associated with P-BE. (XLSX)

S5 Table GSEA results for GO-BP significant categories (corrected p-value<0.05) obtained with the 19 selected genes and 100 GeneMania network neighbors. (XLSX)

S6 Table GSEA results for GO-BP categories (corrected p-value<0.05) obtained with CYR61, TAZ and 100 GeneMania network neighbors. (XLSX)
