# Supplementary material for: CYR61 and TAZ Upregulation and Focal Epithelial to Mesenchymal Transition May Be Early Predictors of Barrett’s Esophagus Malignant Progression
Source: PLoS One. 2016 Sep 1;11(9):e0161967. doi: 10.1371/journal.pone.0161967 (PMC5008832; doi:10.1371/journal.pone.0161967)
Supplement: S1 Fig — (PDF) [file pone.0161967.s001.pdf]

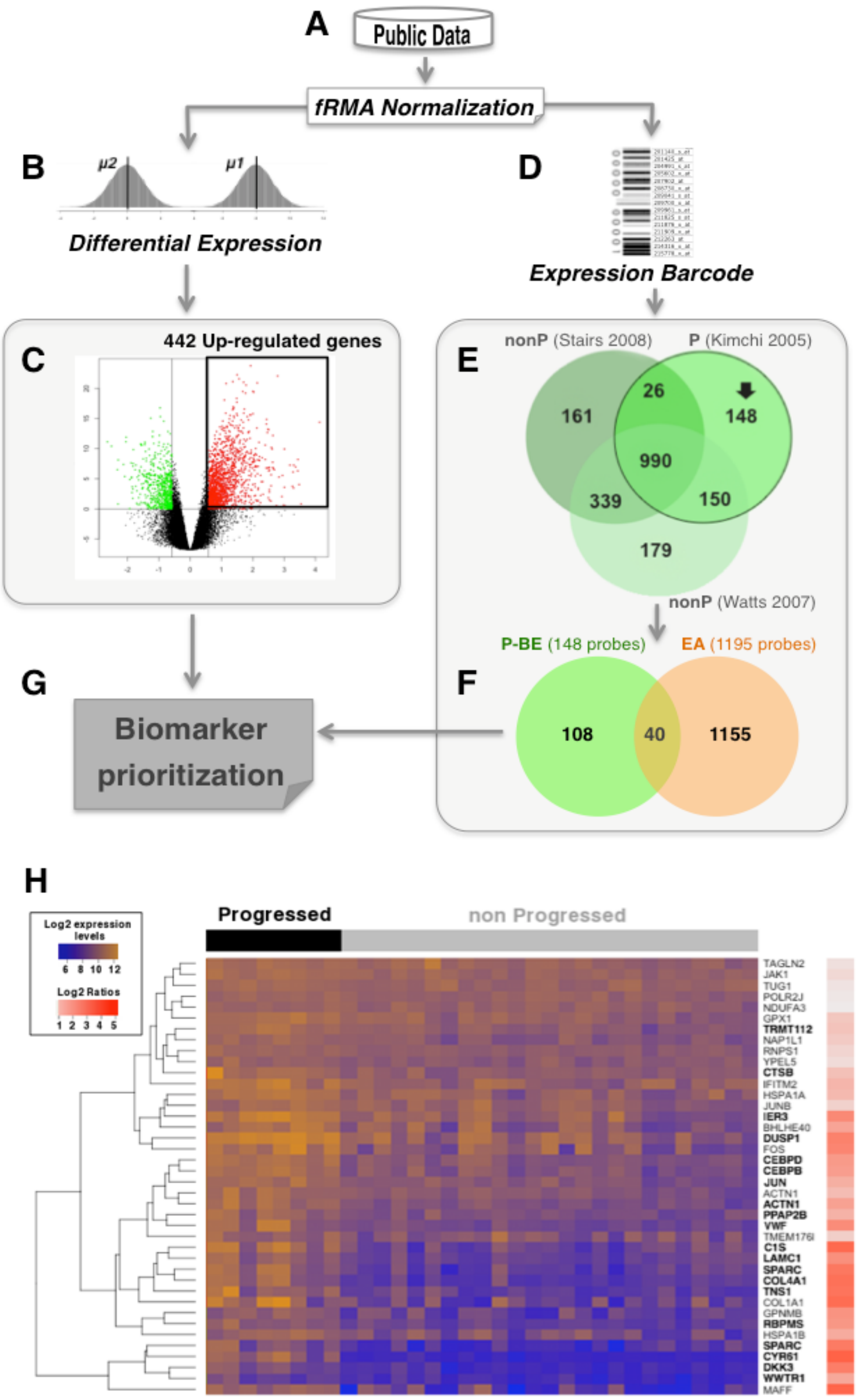

S1 Fig. An original bioinformatics prioritization framework allowed the identification of at risk BE biomarkers. **A.** Publicly available microarray datasets on BE and/or EA were

normalized with the fRMA algorithm. BE samples associated with progression to EA were retrieved from Kimchi *et al.* [1] and assigned to the P-BE group and EA-free BE samples were retrieved from Watts *et al.* [2] and Stairs *et al.* [3] and assigned to nonP-BE group. **B.** Comparison of P-BE versus nonP-BE samples with a differential expression approach which, **C.** resulted in the identification of 442 unique up-regulated genes in P-BE. **D.** Calculation of expression barcodes was performed per individual sample. **E.** P-BE-exclusive probe sets (148) were defined via intersection of P-BE and nonP-BE barcodes. **F.** Forty malignancy-associated progression probe sets were achieved by crossing 148 P-BE barcoded probe sets with EA-associated barcodes. **G.** Integration of barcode and differential expression results to further trim promising candidates for downstream validation. **H.** Combined barcode and differential expression prioritized 20 probe sets associated with BE malignant progression to be validated in downstream experimental settings. In total, 19 unique up-regulated genes (in bold) differentiate P-BE from nonP-BE.

References

1. Kimchi ET, Posner MC, Park JO, Darga TE, Kocherginsky M, Karrison T, et al. Progression of Barrett's metaplasia to adenocarcinoma is associated with the suppression of the transcriptional programs of epidermal differentiation. Cancer Res. 2005;65(8):3146-54. Epub 2005/04/19. doi: 65/8/3146 [pii]
10.1158/0008-5472.CAN-04-2490. PubMed PMID: 15833844.

2. Watts GS, Tran NL, Berens ME, Bhattacharyya AK, Nelson MA, Montgomery EA, et al. Identification of Fn14/TWEAK receptor as a potential therapeutic target in esophageal adenocarcinoma. Int J Cancer. 2007;121(10):2132-9. Epub 2007/06/28. doi: 10.1002/ijc.22898. PubMed PMID: 17594693.

3. Stairs DB, Nakagawa H, Klein-Szanto A, Mitchell SD, Silberg DG, Tobias JW, et al. Cdx1 and c-Myc foster the initiation of transdifferentiation of the normal esophageal squamous epithelium toward Barrett's esophagus. PLoS One. 2008;3(10):e3534. Epub 2008/10/28. doi: 10.1371/journal.pone.0003534. PubMed PMID: 18953412.
