# Supplementary material for: CYR61 and TAZ Upregulation and Focal Epithelial to Mesenchymal Transition May Be Early Predictors of Barrett’s Esophagus Malignant Progression
Source: PLoS One. 2016 Sep 1;11(9):e0161967. doi: 10.1371/journal.pone.0161967 (PMC5008832; doi:10.1371/journal.pone.0161967)
Supplement: S2 Fig — (PDF) [file pone.0161967.s002.pdf]

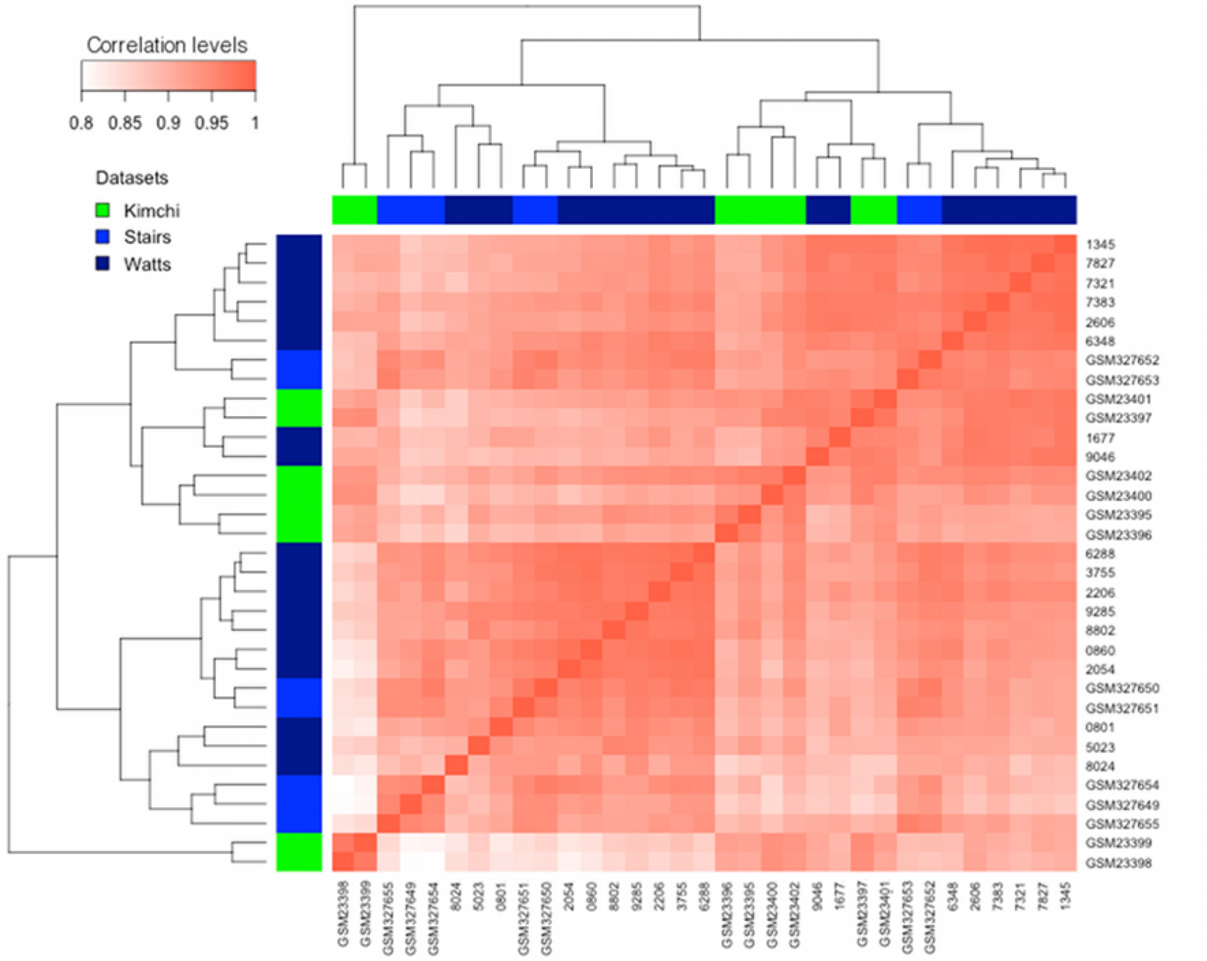

S2 Fig. Microarray data of BE samples from distinct datasets is highly correlated. Pearson's correlations plotted in the heatmap were calculated using fRMA normalized absolute intensity levels and samples were compared through pairwise comparison of all 33 BE samples. Each dataset is represented with a different colour in the coloured side bars.
