# Supplementary material for: CYR61 and TAZ Upregulation and Focal Epithelial to Mesenchymal Transition May Be Early Predictors of Barrett’s Esophagus Malignant Progression
Source: PLoS One. 2016 Sep 1;11(9):e0161967. doi: 10.1371/journal.pone.0161967 (PMC5008832; doi:10.1371/journal.pone.0161967)
Supplement: S3 Fig — (PDF) [file pone.0161967.s003.pdf]

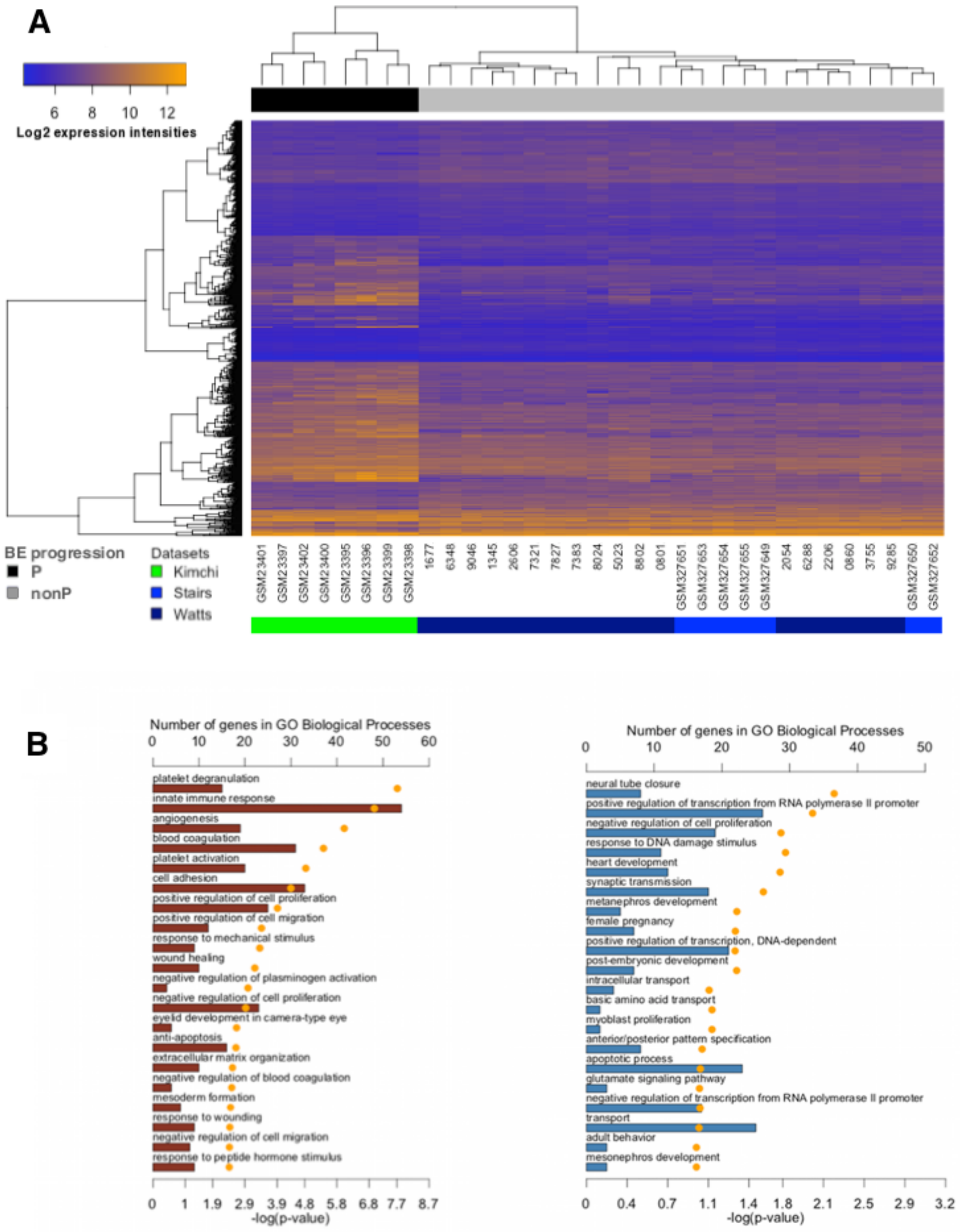

S3 Fig. Differential expression analysis of P-BE and nonP-BE microarray data highlighted more than 700 genes potentially involved in BE malignant progression. A. Heatmap of genes filtered after differential expression analysis with a conservative cutoff (Lods≥5). Each dataset is represented with a different colour in the bottom coloured side bar

and each BE progression group is represented by the upper coloured bar (black and grey). **B.** Top 20 GO Biological Processes (q-value<0.05) over-represented among significantly up-regulated (left plot, in red) and down-regulated (right plot, in blue) genes. Yellow dots illustrate the adjusted Ps for each GO category.
