# Supplementary material for: CYR61 and TAZ Upregulation and Focal Epithelial to Mesenchymal Transition May Be Early Predictors of Barrett’s Esophagus Malignant Progression
Source: PLoS One. 2016 Sep 1;11(9):e0161967. doi: 10.1371/journal.pone.0161967 (PMC5008832; doi:10.1371/journal.pone.0161967)
Supplement: S4 Fig — (PDF) [file pone.0161967.s004.pdf]

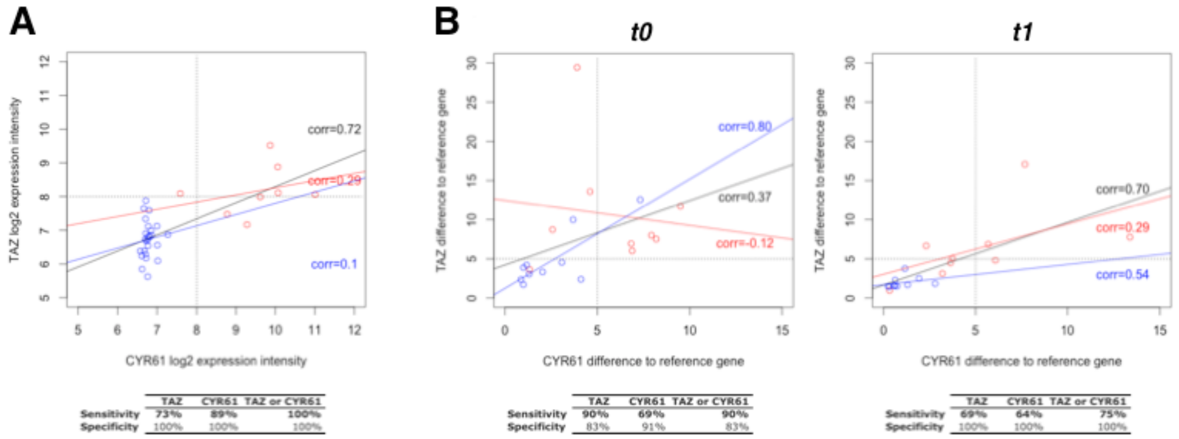

S4 Fig. CYR61 and TAZ expression levels are not correlated. A. Microarray data correlation of CYR61 and TAZ log2 expression intensities. **B.** Correlation of RT-qPCR relative expression levels of CYR61 and TAZ in the index biopsies (t0) and in the recent samples (t1). Results related with P-BE and nonP-BE samples are represented in red and blue, respectively. Black represents results with all samples. A preliminary overview of sensitivity and specificity values for individual or combined usage of biomarkers of at risk BE is shown below each plot.
