# Supplementary material for: CYR61 and TAZ Upregulation and Focal Epithelial to Mesenchymal Transition May Be Early Predictors of Barrett’s Esophagus Malignant Progression
Source: PLoS One. 2016 Sep 1;11(9):e0161967. doi: 10.1371/journal.pone.0161967 (PMC5008832; doi:10.1371/journal.pone.0161967)
Supplement: S5 Fig — (PDF) [file pone.0161967.s005.pdf]

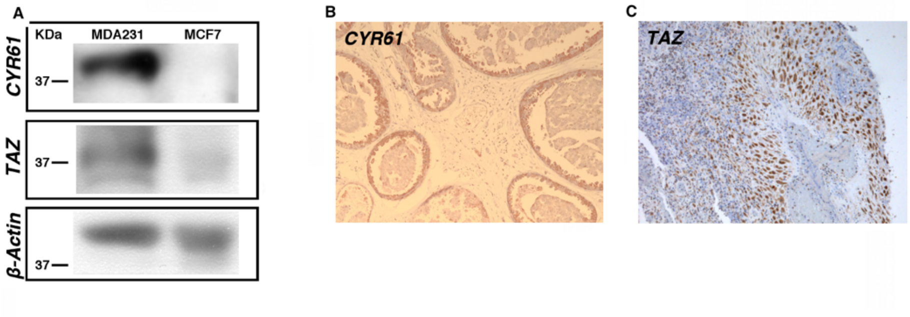

S5 Fig. Validation of CYR61 and TAZ antibodies specificity. A. Western-blot analysis was performed to test the CYR61 and TAZ antibodies specificity on the breast cancer cell lines MDA213 and MCF7. A band of ~42KDa and ~40KDa was observed when using CYR61 and TAZ antibodies, respectively. β–Actin antibody (~50 KDa) was used as a loading control. **B.** Breast cancer and **C.** placenta tissue sections used as used as positive controls of CYR61 and TAZ antibodies, respectively. Magnification 100X.
