# Supplementary material for: CYR61 and TAZ Upregulation and Focal Epithelial to Mesenchymal Transition May Be Early Predictors of Barrett’s Esophagus Malignant Progression
Source: PLoS One. 2016 Sep 1;11(9):e0161967. doi: 10.1371/journal.pone.0161967 (PMC5008832; doi:10.1371/journal.pone.0161967)
Supplement: S6 Fig — (PDF) [file pone.0161967.s006.pdf]

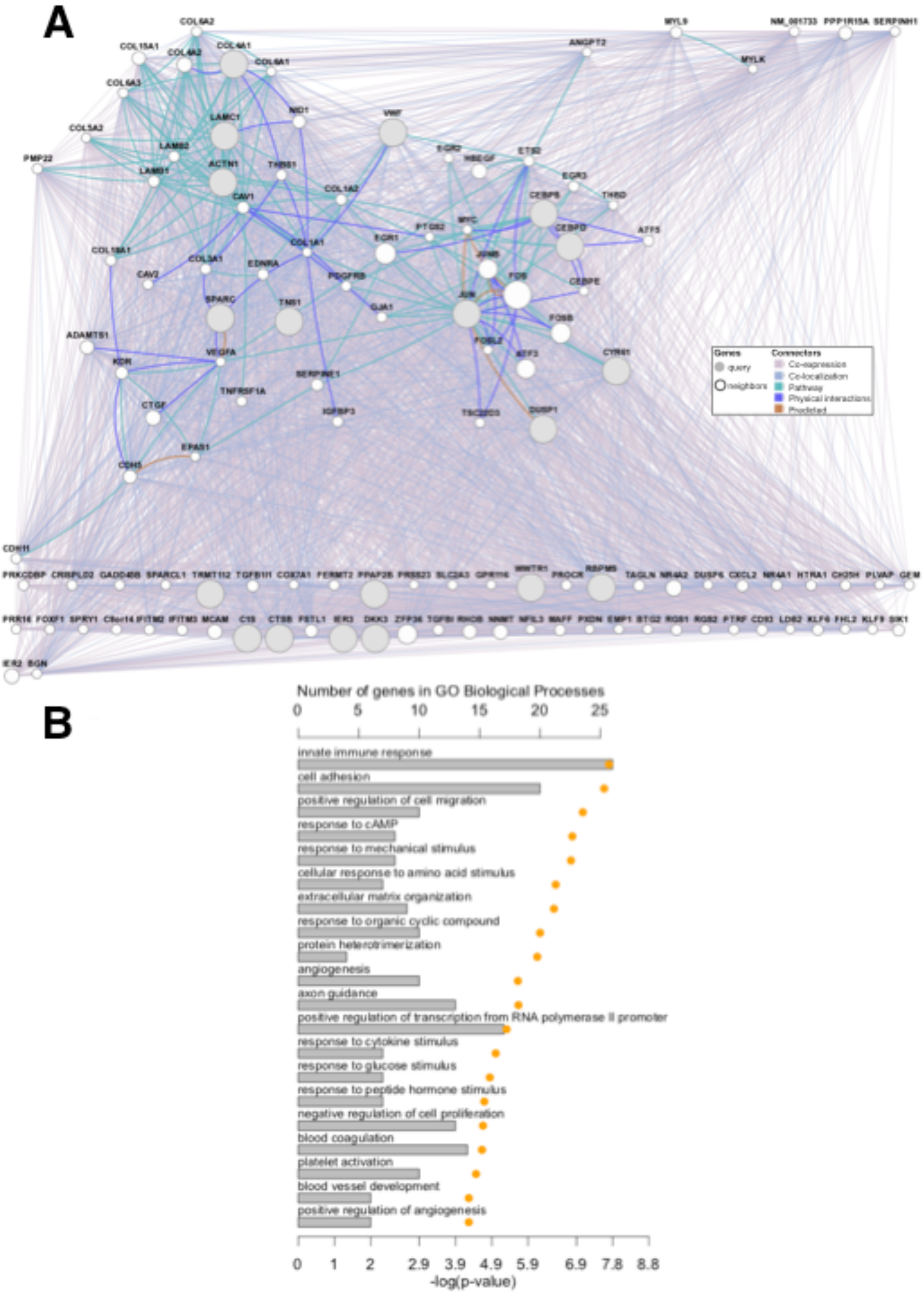

S6 Fig. GeneMania network of the 19 selected candidate biomarkers, network neighbors and associated function showed an enrichment of genes related with cell adhesion, moπ□ptility and response to stimuli. A. The set of 19 genes was use to find

connections with close neighbors according to GeneMania association network algorithm. **B.** Top 20 significant (q-value<0.05) GO Biological Processes were identified by gene set enrichment analysis performed with InnateDB tool. Yellow dots illustrate the adjusted Ps for each GO category.
