# Supplementary material for: CYR61 and TAZ Upregulation and Focal Epithelial to Mesenchymal Transition May Be Early Predictors of Barrett’s Esophagus Malignant Progression
Source: PLoS One. 2016 Sep 1;11(9):e0161967. doi: 10.1371/journal.pone.0161967 (PMC5008832; doi:10.1371/journal.pone.0161967)
Supplement: S7 Fig — (PDF) [file pone.0161967.s007.pdf]

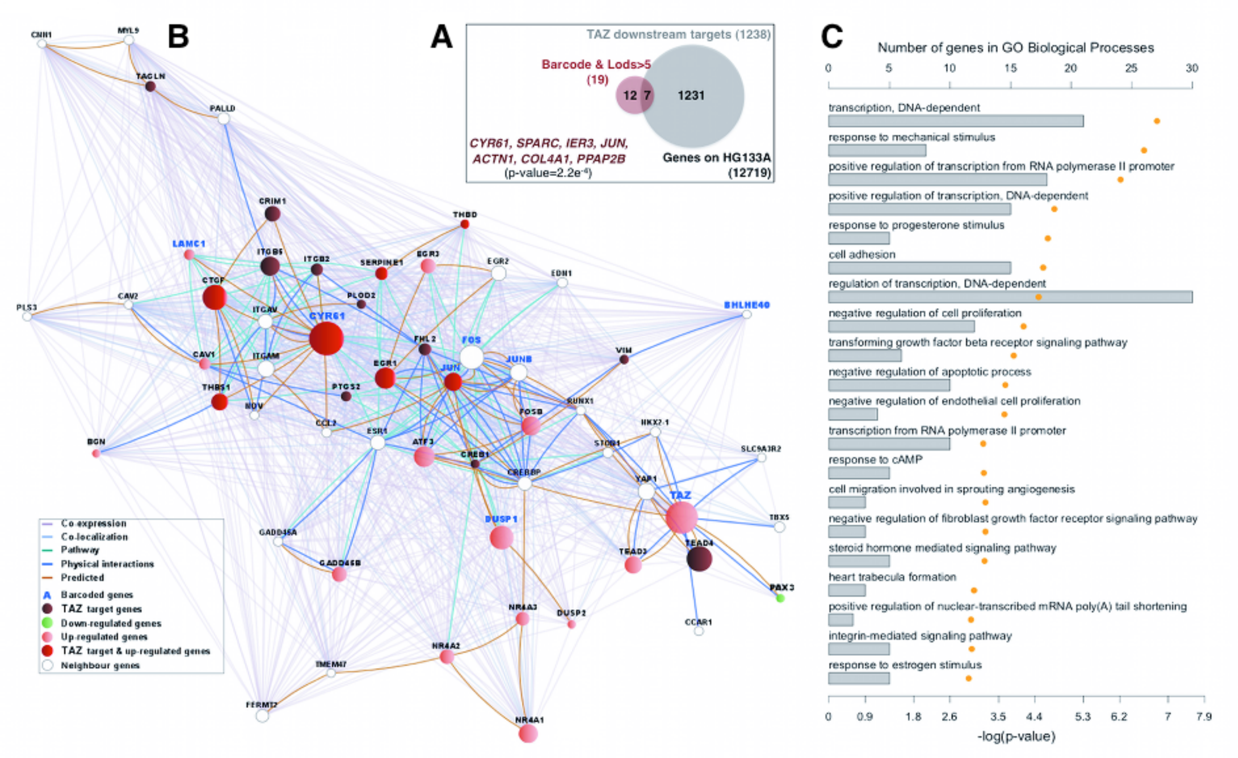

S7 Fig. CYR61 and TAZ are biological linked and share important cellular functions. **A.** TAZ downstream targets represent a category of significantly over-represented genes among the 19 barcode and differentially expressed genes (hypergeometric test pvalue=2.2×10^{-4}, 12719 unique genes represented in the Affymetrix HG-133A GeneChip® platform). **B.** CYR61 and TAZ as query genes in GeneMania network are able to highlight network neighbors which are also TAZ downstream targets, differentially expressed genes and barcode genes. Network genes only connected via co-expression are represented in fig. S4. **C.** Top 20 significantly enriched (q-value<0.05) GO Biological Processes associated with CYR61, TAZ and their 100 GeneMAnia network neighbors. Yellow dots illustrate the adjusted p-values for each GO-BP category.
