# Supplementary material for: CYR61 and TAZ Upregulation and Focal Epithelial to Mesenchymal Transition May Be Early Predictors of Barrett’s Esophagus Malignant Progression
Source: PLoS One. 2016 Sep 1;11(9):e0161967. doi: 10.1371/journal.pone.0161967 (PMC5008832; doi:10.1371/journal.pone.0161967)
Supplement: S1 Results — (PDF) [file pone.0161967.s008.pdf]

Supplementary Results

CYR61 and *TAZ* upregulation and focal epithelial to mesenchymal transition may be early predictors of Barrett's esophagus malignant progression

Joana Cardoso, Marta Mesquita, António Dias Pereira, Mónica Bettencourt-Dias, Paula Chaves, José B. Pereira-Leal

Two main reasons motivated us to develop a new bioinformatics pipeline to search for early biomarkers of BE malignant progression using publicly available transcriptome microarray data. First, while several datasets on BE are in the public domain, none of them simultaneously contained samples from EA, P-BE and nonP-BE that could be directly compared. This issue required us to merge the existing samples from distinct datasets and compare them by DEA. However, inter-dataset DEA is very sensitive to technical noise. Methods such as ComBat and SVA remove batch-associated noise (reviewed in (1)) but due to the reduced number of available BE samples these methods also removed most of the meaningful biological signal. To better deal with the inter-dataset noise and to take advantage of genes with bimodal expression, which are in principle more easily translated to protein level differences and thus the ideal biomarker candidates, we included the Gene Expression Barcode 2.0 binarization algorithm developed by McCall *et al.* (2, 3). The produced barcodes are very robust against random sources of noise because their calculation relies on the usage of a large amount of annotated public data as a reference to binarize the expression of each gene (1=expressed, 0=not expressed) per individual sample.

In the context of our pipeline framework (S1 Fig), we curated three publicly available datasets of BE data on the Affymetrix HG-133A GeneChip® microarray platform. Overall, these three datasets contained a total of 33 BE samples. Samples described as collected in the

context of a clinically diagnosed EA (4) were assigned to the P-BE group (n=8) and samples not associated with EA at the time of analysis (5, 6) were assigned to the nonP-BE group (n=25). After frozen robust multi-array (fRMA) sample normalization (2, 3) we verified that individual samples in the merged set displayed highly correlated expression profiles (Parson's correlation mean=0.92, min=0.80) (S2 Fig). This is indicative that despite the three distinct data sources and associated batch noise, the biological signal of BE samples was very comparable.

We next identified differential gene expression (DGE) between P-BE and nonP-BE samples using a Bayesian DEA, as illustrated in S1 Fig C. Under the very conservative statistical criteria logarithm of the odds (Lods)≥5, probability DGE>99.33%) and a false discovery rate (FDR) of 3.9×10-5 for DGE, we identified 958 independent probe sets mapping to 799 unique ENTREZ id genes (S3 Table). Among the unique genes, we have found up-regulation for 442 (S1 Fig C) and down-regulation for 357 genes. The 799 genes are able to correctly segregate P-BE from nonP-BE samples (S3 Fig A). As anticipated (see Materials and Methods section), no significant probe sets were found after testing of EA samples across distinct datasets (Kimchi *et al.* (4) vs. Watts *et al.* (6)). Barcode binarization of fRMA normalized BE and EA data (S1 Fig D) and the subsequent intersection of P-BE and nonP-BE barcodes allowed us to find a set of 148 probe sets (S4 Table) expressed in the P-BE samples (barcode=1) but oppositely marked as non-expressed (barcode=0) in the nonP-BE samples (S1 Fig E). To find candidates more likely associated with malignancy we assumed that P-BE-specific probe sets should overlap with probe sets expressed in EA (barcode=1). Thus, we have next intersected the 148 and 1195 probe sets, respectively from P-BE and EA barcodes. This procedure resulted in the filtering of malignancy-linked barcode candidates to 40 probe sets, corresponding to 38 unique genes (S1 Fig F), up-regulated in P-BE and EA samples as compared to nonP-BE samples. To maximize the discovery of BE early

progression biomarkers that most likely could be translated into routine clinical practice, we defined our top candidates as genes up-regulated according to DGE results and with barcode values set to 1 (S1 Fig G). With this final step we slimed down the final list of candidates to 20 probe sets, corresponding to 19 unique genes over-expressed in P-BE (S1 Fig H).

Systems biology approach for biomarker prioritization

To improve biomarker prioritization we thought of integrating biological functions of filtered genes with functions potentially relevant for BE malignant progression, by uncovering the gene GO-BP categories over-represented among the 19 filtered biomarkers. First, we used a guilt-by-association GeneMANIA tool (7) to build a functional association network between the set of 19 genes and 100 network neighbors (S6 Fig A). Secondly we used all network players to evaluate the enriched GO-BP categories by GSEA. We have used this alternative strategy instead of directly applying GSEA to the set of 19 genes due its reduced number. GSEA on GeneMania network genes (biomarkers and neighbors) highlighted that the significant (FDR<0.05) GO-BP top categories related with cell adhesion/motility, inflammation, differentiation/wounding, vasculature development, extracellular-matrix and response to stimulus among others (S6 Fig B, S5 Table).

To further increase the odds of success of downstream validation efforts, we have used knowledge-driven biomarker prioritization criteria. To be included, candidates must be functionally linked to 1) top biological functions detected by GSEA and to 2) phenotype features that characterizes BE (e.g. differentiation/wounding responses) and finally 3) candidates must have been previously associated to cancer progression in other tumors. Thus, we have searched the literature for functional characterization of the set of 19 genes and selected two potential biomarkers for proof-of-principle experimental validation. CYR61 (alias CCN1) was the most significantly over-expressed gene in our DGE analysis (S3 Table) and according to barcode analysis is expressed in >93 % of EA samples. Its over-expression

is involved in the malignant progression and prognosis of major tumors (breast, prostate, colorectal and others outlined in Table 1). CYR61 was recently identified in the context of breast cancer (8) as a downstream target of WWTR1 (alias TAZ), one of the barcode and differentially expressed genes. TAZ up-regulation is also implicated in the progressive phenotype of malignant tumors such as breast, colorectal and glioma, among others (Table 1) and was expressed according to barcode in 87% of the EA tumors in our dataset. In addition to CYR61, six other TAZ downstream target genes (SPARC, IER3, JUN, ACTN1, COL4A1, PPAP2B) were significantly enriched (*P*-value=2.2×10-4) among our group of 19 candidates (S7 Fig A), suggesting that specific pathways where CYR61, TAZ and likely other functionally-linked genes operate are deregulated during BE-associated EA progression. To further test this functional link hypothesis we explored CYR61 and TAZ interacting genes with gene GeneMania networking algorithm (S7 Fig B). Among network genes we identified barcode genes (e.g. FOS, JUN, LAMC1), significantly up-regulated genes (e.g. TEAD3, FOSB, ATF3) of which some are TAZ downstream targets (e.g. CTFG, JUN, EGR1). Analysis of top GO-BP categories (FDR<0.01) over-represented among CYR61, TAZ and neighbors (S7 Fig C) pointed to biological functions involving cell adhesion/migration, transcription and response to stimulus (S6 Table). One hypothesis suggested by the data is that P-BE samples have deregulated transcriptional responses to diverse stimuli, including an up-regulation of cell adhesive and migratory properties which will ultimately contribute to the malignant phenotype of BE cells.

References

1. Chen C, Grennan K, Badner J, *et al.* Removing batch effects in analysis of expression microarray data: an evaluation of six batch adjustment methods. *PLoS One* 2011; 6(2): e17238.

2. McCall MN, Uppal K, Jaffee HA, Zilliox MJ, Irizarry RA. The Gene Expression Barcode: leveraging public data repositories to begin cataloging the human and murine transcriptomes. *Nucleic Acids Res* 2011 Jan; 39(Database issue): D1011-5.
3. Zilliox MJ, Irizarry RA. A gene expression bar code for microarray data. *Nat Methods* 2007 Nov; 4(11): 911-3.
4. Kimchi ET, Posner MC, Park JO, *et al.* Progression of Barrett's metaplasia to adenocarcinoma is associated with the suppression of the transcriptional programs of epidermal differentiation. *Cancer Res* 2005 Apr 15; 65(8): 3146-54.
5. Stairs DB, Nakagawa H, Klein-Szanto A, *et al.* Cdx1 and c-Myc foster the initiation of transdifferentiation of the normal esophageal squamous epithelium toward Barrett's esophagus. *PLoS One* 2008; 3(10): e3534.
6. Watts GS, Tran NL, Berens ME, *et al.* Identification of Fn14/TWEAK receptor as a potential therapeutic target in esophageal adenocarcinoma. *Int J Cancer* 2007 Nov 15; 121(10): 2132-9.
7. Montojo J, Zuberi K, Rodriguez H, *et al.* GeneMANIA Cytoscape plugin: fast gene function predictions on the desktop. *Bioinformatics* 2010 Nov 15; 26(22): 2927-8.
8. Zhang H, Liu CY, Zha ZY, *et al.* TEAD transcription factors mediate the function of TAZ in cell growth and epithelial-mesenchymal transition. *J Biol Chem* 2009 May 15; 284(20): 13355-62.
